# Biorefining Brazilian Green Propolis: An Eco-Friendly Approach Based on a Sequential High-Pressure Extraction for Recovering High-Added-Value Compounds

**DOI:** 10.3390/molecules30010189

**Published:** 2025-01-06

**Authors:** Guilherme Dallarmi Sorita, Wilson Daniel Caicedo Chacon, Monique Martins Strieder, Camilo Rodriguez-García, Alcilene Monteiro Fritz, Silvani Verruck, Germán Ayala Valencia, José A. Mendiola

**Affiliations:** 1 Foodomics Laboratory, Institute of Food Science Research (CIAL) (CSIC-UAM), Nicolás Cabrera 9, 28049 Madrid, Spain; 2 Department of Chemical and Food Engineering, Federal University of Santa Catarina, Florianópolis 88040-900, Santa Catarina, Brazil; 3 Multidisciplinary Laboratory of Food and Health (LabMAS), School of Applied Sciences (FCA), Universidade Estadual de Campinas, Campinas 13484-350, São Paulo, Brazil; 4 High Pressure Laboratory, Food Chemistry Research Group, Departamento de Química, Facultad de Ciencias, Universidad Nacional de Colombia, Carrera 30 No. 45-03, Bogotá D.C. 111321, Colombia; 5 Department of Food Science and Technology, Federal University of Santa Catarina, Rodovia Admar Gonzaga, 1346, Itacorubi, Florianópolis 88034-000, Santa Catarina, Brazil

**Keywords:** process optimization, bio-based solvent, phytochemicals, environmental impact, natural products

## Abstract

Propolis is a valuable natural resource for extracting various beneficial compounds. This study explores a sustainable extraction approach for Brazilian green propolis. First, supercritical fluid extraction (SFE) process parameters were optimized (co-solvent: 21.11% *v*/*v* CPME, and temperature: 60 °C) to maximize yield, total phenolic content (TPC), antioxidant capacity, and LOX (lipoxygenase) inhibitory activity. GC–MS analysis identified 40 metabolites in SFE extracts, including fatty acids, terpenoids, phenolics, and sterols. After selecting the optimum SFE process parameters, a sequential high-pressure extraction (HPE) approach was developed, comprising SFE, pressurized liquid extraction (PLE) with EtOH/H_2_O, and subcritical water extraction (SWE). This process was compared to a similar sequential extraction using low-pressure extractions (LPE) with a Soxhlet extractor. The HPE process achieved a significantly higher overall yield (80.86%) than LPE (71.43%). SFE showed higher selectivity, resulting in a lower carbohydrate content in the non-polar fraction, and PLE extracted nearly twice the protein amount of LPE–2. Despite the HPE selectivity, LPE extracts exhibited better acetylcholinesterase (AChE), butyrylcholinesterase (BChE), and LOX inhibition, demonstrating that the neuroprotective and anti-inflammatory activity of the extracts may be associated with a symbiosis of a set of compounds. Finally, a comprehensive greenness assessment revealed that the HPE process proved more sustainable and aligned with green chemistry principles than the LPE method.

## 1. Introduction

Propolis, a complex resin crafted by bees, is rich in phenolics, flavonoids, terpenoids, and other bioactive compounds with diverse biological properties, such as antimicrobial, antioxidant, and anti-inflammatory attributes [1]. Supported by extensive research and clinical trials, propolis has found widespread application across various sectors, including medicine, cosmetics, aromatherapy, food, and veterinary science [1,2,3,4].

Extraction processes are essential for isolating phytochemicals from natural materials, such as propolis, to develop innovative ingredients. Modern extraction methods aim to replace traditional, environmentally harmful techniques [5,6]. In this context, high-pressure techniques like supercritical fluid extraction (SFE), pressurized liquid extraction (PLE), and subcritical water extraction (SWE) appear as effective and eco-friendly methods to extract natural molecules from propolis [7,8].

Previous studies on green propolis extraction focused primarily on recovering bioactive compounds using low- and high- extraction methods, but often neglected the sustainability of the extraction process [3,9,10,11]

Biorefinery systems integrating SFE, PLE, and SWE into an exhaustive extraction approach can recover a broader range of bioactive compounds [12,13]. Furthermore, a detailed sustainability assessment is crucial, as it addresses both extraction efficiency and environmental impact and is aligned with sustainable development goals.

Sustainability is crucial in propolis extraction, since it enhances existing processes by promoting eco-friendly practices, reducing resource use, minimizing waste, and encouraging the use of renewable resources, all of which ensure the long-term growth and sustainability of the green chemistry industry [14,15].

In line with this, the present work proposed a new sustainable extraction approach for recovering biomolecules from Brazilian green propolis. The present study investigates the effectiveness of the sequential high-pressure extraction (HPE) process in recovering biomolecules from Brazilian green propolis compared to low-pressure extraction (LPE), evaluating the impact of these methods on the yield and bioactivity of the extracts and sustainability.

For this, an HPE process was proposed involving three steps: (i) SFE with the optimized parameters, (ii) PLE using EtOH/H_2_O, and (iii) SWE with water as the solvent. For comparison, the same sequential extraction approach was carried out with LPE. Extracts from both sequential processes were evaluated for total phenolic content (TPC), total flavonoid content (TFC), carbohydrate and protein content, antioxidant activity (ABTS and DPPH), neuroprotective effects (AChE and BChE inhibition), and anti-inflammatory effects (LOX inhibition) to identify potential applications for the different fractions. Finally, a greenness assessment using the *Path2green* model was conducted to compare the sustainability of HPE and LPE.

## 2. Results

### 2.1. SFE Optimization

Prior to FFD optimization, a kinetic study was conducted, before optimizing SFE at 20% CPME and 50 °C (central point of FFD) to determine the optimal extraction time. Figure S1 (Supplementary Materials) shows the kinetic curve obtained during 60 min of extraction. The kinetic curve showed a rapid yield increase during the first 20 min, reaching about 40 g 100 g^−1^ of propolis (conventional mechanism). From 20 to 40 min, the yield increased slowly to 50 g 100 g^−1^, reflecting decreased efficiency as accessible compounds were depleted (a combination of convection and diffusion mechanisms). After 30 min, the process was primarily controlled by diffusion, leading to minimal yield increases. Therefore, the optimal extraction time was maintained at 30 min, recovering 45 g 100 g^−1^ of extractable substance.

**Figure 1:**
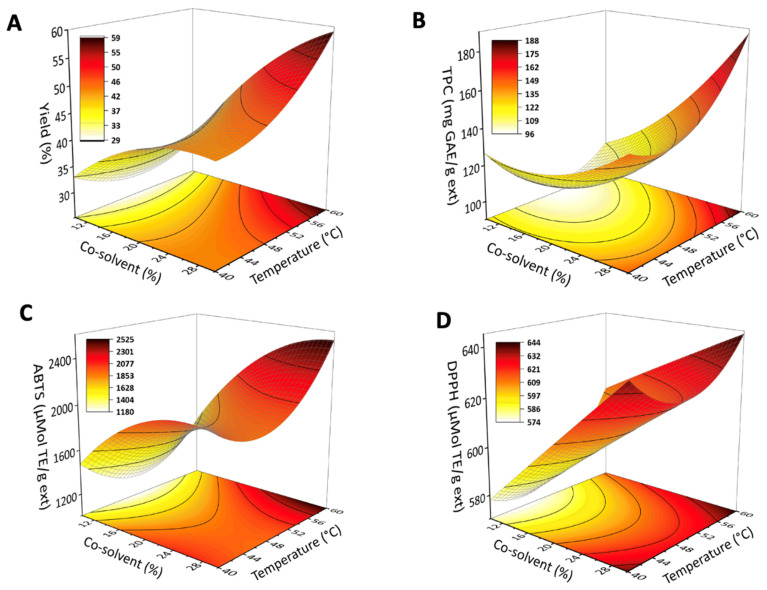
Response surfaces of SFE of bioactive compounds from propolis. Studied factors: temperature and co-solvent percentage on the (**A**) extraction yield, (**B**) TPC, (**C**) ABTS, and (**D**) DPPH. Extraction pressure 20 MPa and flow 4 mL min^−1^ constant in all experiments.

After determining the optimal extraction time, the FFD experimental design was conducted to optimize the key SFE parameters: temperature and co-solvent proportion. Table S1 summarizes the effects of temperature (40, 50, and 60 °C) and CPME percentage (10, 20, and 30%) in CO_2_ on the extraction yield, TPC, ABTS, DPPH, ABTS, and LOX results. Figure S2 (Supplementary Materials) and Figure 1 provide a clearer understanding of the effects of each experimental parameter and their interactions, through standardized Pareto charts and surface response plots, respectively.

**Figure 2:**
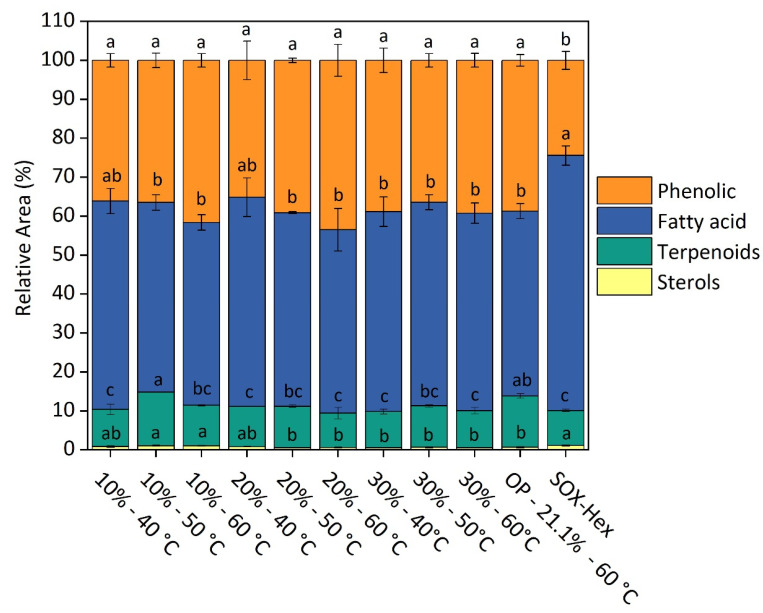
Relative abundance (%) of phenolic compounds, fatty acids, terpenoids, and sterols of SFE extracts, optimum point, and SOX-Hex determined by GC–MS. Legend: Fatty acids: sum of the relative abundance (%) of hexadecanoic acid–palmitic acid, octadecanoic acid–stearic acid, 9,12-Octadecadienoic acid–linoleic acid, alpha-linolenic acid, alpha-linolenic acid isomer, alpha-linolenic acid isomer 2, 9,12-Octadecadienoic acid isomer 2, 11-Eicosenoic acid–omega 9, pentanoic acid, pentanoic acid–valeric acid isomer, hexadecanoic acid–monopalmitin, 5-Eicosene, (E)-, octadecanoic acid–Stearic acid, tetracosanoic acid–lignoceric acid, tetracosanoic acid isomer, cis-5,8,11-eicosatrienoic acid, and decanoic acid. Terpenoids: sum of the relative abundance (%) of trans-Caryophyllene, trans-Caryophyllene isomer, phenol, 2,6-di-tert-butylphenol, alpha-Copaene, nerolidol isomer, (+) spathulenol, (−)-Caryophyllene oxide, trans–trans-farnesol, pseduosarsasapogenin-5,20-dien, trans, trans-farnesol isomer, pentitol, farnesol, myrtenol, tetrahydro linalool, and globulol. Sterols: relative abundance (%) of ergost-25-ene-3,5,6,12-tetrol). Phenolic compounds: sum of the relative abundance (%) of benzenepropanoic acid, hydrocinnamic acid, cinnamic acid, 1,3-Benzenedicarboxylic acid, and trans-Aconitic acid. OP: optimized SFE extract. Means followed by the different letters in the bars indicate a significant statistical difference by Tukey’s test (*p* ≤ 0.05).

Furthermore, a comprehensive lipid profiling analysis of the extracts obtained in SFE process optimization (Table S1) and Soxhlet extraction with hexane (SOX-Hex) samples was conducted using GC–MS to support the discussion of the yield, phenolic content, antioxidant activities (ABTS and DPPH), and LOX inhibitory activity model prediction. Table S7 (Supplementary Materials) shows retention time, the family of each compound, formula, match factor, monoisotopic mass, and fragmentation patterns. Figure 2 illustrates the relative abundance of key bioactive compounds, including phenolic compounds, fatty acids, terpenoids, and sterols, extracted from Brazilian green propolis through optimized SFE and Soxhlet extraction with hexane (SOX-Hex). The figure highlights the differences in the chemical profiles of the extracts obtained via these two methods, demonstrating the higher ability of SFE to extract a higher proportion of phenolic compounds and terpenoids, which are known for their potent antioxidant and anti-inflammatory properties. In contrast, the SOX-Hex extraction resulted in a greater abundance of fatty acids, reflecting the limited solubility range of this conventional method compared to the broader capabilities of the optimized SFE process. These findings underscore the selectivity and efficiency of SFE in targeting a wide range of valuable bioactive compounds from propolis.

To better understand the factors influencing yield, the predicted model for yield, summarized in Table S2, reveals that co-solvent (A) and temperature (B) exert a significant influence on yield (*p*-value < 0.05). Furthermore, the interaction between co-solvent percentage and temperature (AB) was also statistically significant (*p*-value = 0.039), suggesting a synergistic effect between these factors on the response variable. The SFE process parameters of 20–60 °C and 30–60 °C yielded the highest extraction rates, with values of 59% and 51%, respectively. Under these conditions, SFE exceeded SOX-Hex extraction by more than twofold, which presented a yield of 19%. The high yield of SFE justified by the increase in CPME (%) enhanced the solvent polarity, improving the solubility of a wide range of analytes and confirming the clear effect of the co-solvent. These results corroborate the extracted compounds’ profiles presented in Figure 2. The profile of compounds obtained by SOX-Hex presents a higher proportion of fatty acids and a lower proportion of phenolic compounds, indicating the smaller solubility range of this methodology compared with SFE.

Also, the high pressure (20 MPa) and the highest temperature (60 °C) applied in the SFE process significantly increase the solubility of the solvent (in this study, CO_2_ + CPME), enhancing its solvent power and, consequently, its ability to dissolve compounds [8]. Adding CPME as a co-solvent in CO_2_ allowed for higher extraction yields than just supercritical CO_2_, as observed by Fachri et al. [16]. They obtained an extraction yield of 14.4 g 100 g^−1^ from Indonesian *Trigona* sp. propolis employing similar conditions: 50 °C, 250 bar, pure CO_2_ flow rate of 15 g min^−1^, 50 g raw propolis (S/F = 72), and a 240 min dynamic extraction time.

Regarding the TPC model, the evaluation of linear factors indicated that co-solvent percentage substantially impacted TPC (*p*-value = 0.000), while temperature alone was not significant (*p*-value = 0.194, higher than 0.05) (Figure 1B and Table S3). This behavior suggests that the co-solvent played a more crucial role in extracting the TPC, while temperature did not exhibit a strong effect in this context. However, the quadratic term for temperature (*p*-value = 0.015) was also significant, suggesting a non-linear relationship between TPC and temperature (*p*-value = 0.015). Additionally, the interaction between co-solvent percentage and temperature was significant (*p*-value = 0.016), indicating that the impact of the co-solvent varies depending on the temperature. The results indicate that although temperature can aid in extracting phenolic compounds by enhancing their diffusion from the green propolis matrix into the solvent, this effect is generally less pronounced than the influence of the co-solvent increase [17]. For example, by fixing the temperature at 60 °C, it is possible to observe an increase in TPC values with the increase in the co-solvent proportion (10%: 104.33, 20%: 132.36, and 30%: 198.72 mg GAE mL^−1^, Table S1), as depicted in Figure 1B. GC–MS analysis did not detect significant differences in TPC among SFE fractions. However, the observed increase in TPC values with a rising co-solvent proportion suggests that a proportion of the phenolic compounds in these extracts are non-volatile and, thus, not detectable by GC–MS.

SFE extracts from propolis presented outstanding antioxidant capacity. The ABTS method provided antioxidant values ranging from 1346 (10% CPME and 60 °C) to 2703 µmol TE g^−1^ (30% CPME and 60 °C), while DPPH ranged from 579 (10% CPME and 40 °C) to 644 µmol TE g^−1^ (30% CPME and 40 °C) (Table S1). Propolis extracts exhibited higher antioxidant activity when measured by the ABTS assay than the DPPH assay. This difference was attributed to the specificity mechanism of the ABTS method, which detects a broader range of compounds encompassing both polar and non-polar ones. In contrast, DPPH primarily identifies polar antioxidants [18]. Figure 2 shows SFE extracts with a relevant percentage of terpenoid (8.84–13.76%, relative area) and phenolic compounds (35.15–43.47%, relative area), probably with more non-polar characteristics since they were volatile and detected in GC–MS. This high non-polar bioactive compound content explains the higher activity observed by the ABTS method. The use of CPME as a solvent likely contributed to the extraction of a broader range of antioxidants better captured by the ABTS method. The same behavior was observed in the study by Gomes et al. [19], who found ABTS values for ethanolic propolis extracts almost twice as high as DPPH values in propolis from different Brazilian regions.

LOX inhibitory activity was used in this study as an indicator of the anti-inflammatory potential of propolis extracts. However, the temperature (*p*-value = 0.192) and percentage of co-solvent (*p*-value = 0.084) in the SFE did not significantly affect this activity (Table S6). LOX activity of propolis extracts refers to the function of the lipoxygenase enzyme, which plays a role in the metabolism of fatty acids and is linked to inflammatory processes. In this sense, the LOX inhibitory assay measures how effectively a substance can inhibit this enzyme, which is important for reducing inflammation and potentially treating related diseases [20].

The LOX inhibition results in this work were expressed in mg QE mL^−1^, which makes direct comparison with most studies reporting IC_50_ values difficult. However, high anti-inflammatory activity was observed by El-Guendouz et al. [21], with IC_50_ values varying from 0.02 to 0.65 mg mL^−1^ in Moroccan propolis samples. Although LOX inhibition can be related to antioxidant activity, the significant variation in antioxidant activity (Table S1) while LOX inhibition values remained statistically unchanged suggests that the compounds responsible for LOX inhibition might differ from those responsible for the antioxidant capacity. While LOX inhibition and antioxidant activity are valuable for potential therapeutic applications, they are not necessarily correlated. Several studies suggest the potential anti-inflammatory activity of terpenoids [21,22,23].

In the present study, terpenoid content (GC–MS results, Figure 2) in SFE extracts varied from 8.85 to 13.79%. Among the terpenoids identified in SFE extracts, 2,6-di-tert-butyl phenol was the predominant terpenoid identified in SFE extracts, accounting for 1.90–3.37% of the relative area (Figure 2). This compound is known for its antioxidant properties, attributed to its bulky tert-butyl groups, enabling it to scavenge free radicals. Studies have suggested that 2,6-di-tert-butyl phenol may inhibit lipoxygenase (LOX), potentially contributing to anti-inflammatory activity by preventing the formation of pro-inflammatory leukotrienes [24]. Thus, it could be suggested that 2,6-di-tert-butyl phenol in SFE extracts may contribute (in part) to their observed anti-inflammatory activity.

A multiple response optimization was applied to maximize extraction yield while concurrently maximizing TPC, ABS, DPPH, and LOX values. The predicted and experimental results are outlined in Table 1.

Under the optimized conditions of 21.1% CPME and 60 °C, the process yielded a product with a yield of 55.93%, TPC of 148.92 mg GAE g^−1^, ABTS and DPPH values of 2455.10 and 620.0 µmol TE g^−1^, respectively, and a LOX content of 15.16 mg QE g^−1^. The close agreement between the predicted and experimental values, as shown in Table 1, indicates that the optimization model effectively predicts the responses under the optimum conditions. The small deviation between the predicted and experimental results (e.g., yield of 55.9% vs. 56.7%, TPC of 149 mg GAE g^−1^ vs. 166.7 mg GAE g^−1^) demonstrates the accuracy and reliability of the model. These results suggest a robust optimization approach and can reliably guide the process conditions to achieve the desired outcomes. The small variations observed in some responses are within the experimental error, further confirming the strength of the optimization method in predicting the experimental values.

Moreover, the optimized condition allowed an extract with a higher proportion of terpenoids than the other conditions (Figure 2), which may have allowed the highest activity of the extract obtained under this condition. This extract, rich in fatty acids (47.48%), phenolic compounds (38.72%), terpenoids (13.16%), and sterols (0.64%) could be used in the formulation of various products. Thus, the optimal point parameters were adopted as the starting point for the biorefinery of the HPE process, which will be discussed in the next section.

### 2.2. Biorefining Green Propolis: Sequential High-Pressure Extractions Approach

**Figure 3:**
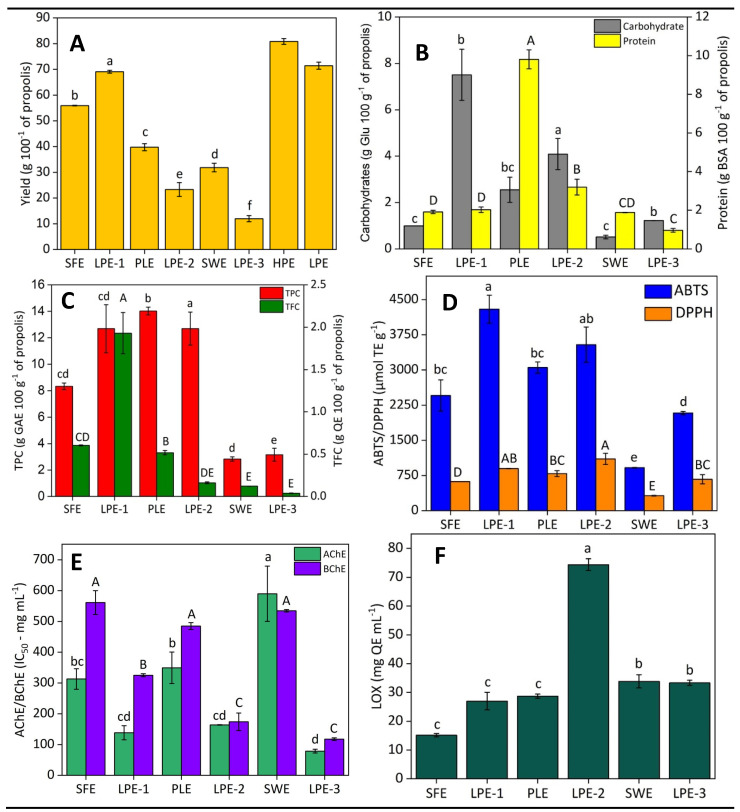
Fractions (**A**) yield (%), (**B**) total carbohydrate and protein content, (**C**) total phenolic content (TPC) and total flavonoid content (TFC), (**D**) antioxidant capacity (ABTS and DPPH methods), (**E**) AChE and BChE inhibitory activities, and (**F**) LOX inhibitory activity. Fractions obtained from green propolis by high-pressure extraction (HPE) and low-pressure extraction (LPE) using the biorefinery approach. See extraction conditions in Figure 4. Means followed by the different letters in the bars indicate a significant statistical difference by Tukey’s test (*p* ≤ 0.05).

Following the biorefinery concept, this study examined the application of sequential high- and low-pressure extraction, and the results of process efficiency and extract quality for each step are depicted in Figure 3.

**Figure 4:**
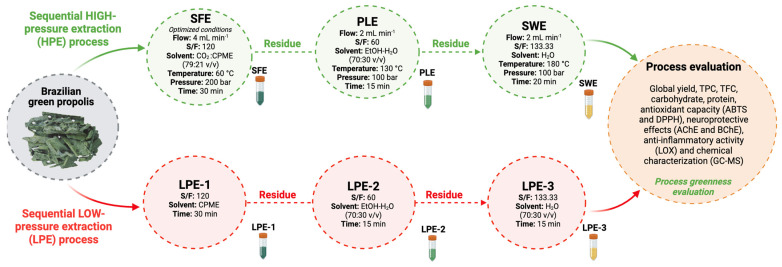
Sequential extraction approach proposed to recover high-added-value compounds from Brazilian green propolis.

#### 2.2.1. First Biorefinery Step: SFE and LPE–1

More than 50% of the raw material was recovered in the first step, 56% with SFE and 69% with LPE–1 using CPME, as depicted in Figure 3A. The identical extraction parameters (time and solid-to-fluid ratio) used in both experiments suggest that the higher yield obtained by LPE–1 can likely be attributed to the use of pure CPME, compared with SFE, which employed CPME as a co-solvent of CO_2_. Pure CPME has a higher boiling point and increased solubility for certain macromolecules, particularly non-polar compounds such as lipids. This highlights the higher initial extraction efficiency of LPE–1 for non-polar components, although the selectivity of SFE towards specific bioactive compounds such as terpenoids and phenolics may offer distinct advantages for certain applications. Given the high oily fraction in the raw material (18.93% oil) in green propolis, it is plausible that this higher yield is related to the extraction of those compounds. Following the extraction of fatty acids, the SFE process also successfully extracted a range of non-polar compounds, including terpenoids, sterols, and phenolic compounds (Figure 2). This demonstrates the versatility of SFE in isolating multiple bioactive compounds within a single extraction step, making it highly suitable for multi-purpose biorefinery applications. Moreover, in LPE–1, the CPME boiling temperature of 106 °C probably increased convective and diffusive rates, compared to CPME as a co-solvent in SFE limited to a maximum temperature of 60 °C.

The results of the total carbohydrate content (Figure 3B) confirm the higher selectivity of the SFE method. SFE yielded an extract almost free of carbohydrates (0.99 g Glu 100 g⁻^1^) compared to LPE–1, which presented a significantly higher carbohydrate content (7.51 g Glu 100 g⁻^1^). Conversely, there was no statistically significant difference in total protein content between the extraction methods, with both SFE (1.91 g 100 g⁻^1^) and LPE–1 (2.03 g 100 g⁻^1^) yielding relatively low protein levels. This selectivity of SFE is advantageous for obtaining specific compounds, making it ideal for producing extracts with minimal carbohydrate content, unlike LPE, which leads to significant carbohydrate extraction. This higher selectivity suggests that SFE could be particularly advantageous for applications where high-purity extracts with minimal carbohydrate interference are required, such as in the nutraceutical industry.

Likely due to the medium polarity of CPME, the first stage of the biorefinery allowed for the production of extracts with high TPC, LPE–1 (12.69 g GAE 100 g^−1^), and SFE (8.33 g GAE 100 g^−1^), but the process type did not have a significant effect on this response (Figure 3C). In this sense, the solvents used (CPME and CO_2_ + CPME) under the conditions employed probably provided similar phenolic compound solubility, providing the same yields. Despite the high presence of fatty acids, the first extraction yielded a significant number of total flavonoids, in which LPE–1 yielded 1.9 g QE 100 g^−1^ and SFE yielded 0.6 g QE 100 g^−1^ (Figure 3C). This statement highlights the effectiveness of using less polar solvents with high boiling points, such as CPME (4.7, 106 °C), for extracting these compounds, even under low-pressure conditions. Phenolic compounds are the main class of natural antioxidants, exerting their antioxidant capacity through different mechanisms [18]. Consequently, the substantial TPC and TFC in the extracts from the first extraction step contributes to remarkable antioxidant activities (Figure 3B), as measured by both the ABTS and DPPH methods. The LPE–1 extract exhibited stronger antioxidant activity, with ABTS activity measured at 4295 μmol TE g^−1^ and DPPH activity at 897 μmol TE g^−1^. In comparison, the SFE extract showed lower values, with an ABTS activity of 2455 μmol TE g^−1^ and DPPH activity of 620 μmol TE g^−1^. These differences may be attributed to the different solubility profiles of the solvents used in LPE–1 and SFE, where LPE–1 may be more effective at extracting more polar antioxidants (compared to SFE), which contribute more significantly to ABTS and DPPH scavenging. Another possibility is that they could be attributed to the combination of higher carbohydrates and phenolics found, so the carbohydrates are probably not free radical scavenging, but linked to phenolic compounds with antioxidant activity. The higher antioxidant activity of the LPE–1 extract suggests that it could be more beneficial in formulations requiring robust free radical scavenging properties, such as cosmetic anti-aging products or nutraceuticals.

The LPE–1 extract demonstrated stronger neuroprotective activity, with significantly lower IC50 values for both AChE (138 μg mL^−1^) and BChE (325 μg mL^−1^) compared to the SFE extract (AChE: 313 μg mL^−1^; BChE: 561 μg mL^−1^). The same behavior was observed for anti-inflammatory activity, for which LPE–1 (26.99 mg QE g^−1^) showed a higher value than SFE (15.16 mg QE g^−1^). These results suggest that the LPE–1 method may facilitate the extraction of a synergistic combination of bioactive compounds, potentially including flavonoids, phenolics, and fatty acids, which contribute to its enhanced enzyme inhibitory capacity. The interaction between antioxidants, anti-inflammatory agents, and enzyme-modulating compounds likely amplifies the neuroprotective effects [25,26,27,28]. Therefore, the enhanced activity observed in the LPE extract could result from this synergy, where the combined action of compounds is more effective than their individual effects. This higher inhibitory activity could make the LPE extracts more suitable for applications targeting neurodegenerative diseases such as Alzheimer’s, where the inhibition of these enzymes is critical.

#### 2.2.2. Second Biorefinery Step: PLE and LPE–2

The second biorefinery step allowed lower extraction yields of 23.31% and 39.78% for LPE–2 and PLE, respectively. This difference may be attributed to the result of the first stage, in which the high-pressure technique (SFE) provided a lower yield than the low-pressure technique (LPE–1). In this biorefinery, the initial step was vital for removing lipids and other CPME-soluble compounds, preparing the matrix for improving the recovery of bioactive compounds like phenolics, flavonoids, and carbohydrates that may be initially trapped or shielded by less polar substances. Additionally, the pressurization and depressurization process during SFE enhances solvent penetration and can rupture cellular structures, aiding in releasing otherwise difficult-to-extract compounds [29]. The LPE–2 approach using ethanol/water extracted substantial amounts of carbohydrates (4.09 g Glu 100 g^−1^) and proteins (3.20 g BSA 100 g^−1^). The PLE extract was characterized by a significantly lower carbohydrate content (2.55 g Glu 100 g^−1^), but achieved a higher recovery of proteins, extracting nearly twice the amount compared to LPE–2 (9.81 g BSA 100 g^−1^). This is likely due to the higher pressure and temperature conditions used in PLE, which enhance the cell wall disruption and solubilization of proteins. The increased protein content in the PLE extract positions it as an ideal candidate for applications in the food industry, particularly in nutraceuticals and functional foods, where protein enrichment is highly valued.

The combined results of step 2 suggest that extraction pressure significantly influences extract composition. The high pressure applied on PLE resulted in a higher protein yield in the extract. The increased pressure aids in breaking down plant cells and solubilizing proteins [30], thereby boosting the overall yield. Observing the global HPE results (Figure 3), it is evident that PLE yielded a higher protein content than LPE–2, which probably contributed to the higher yield but lower antioxidant activity (by the DPPH method) observed in this extract. Proteins can interact with phenolic compounds to form complexes that may protect them from oxidation [31]. This protective effect can paradoxically result in a reduced observed antioxidant capacity of the phenolic compounds in the extract, as part of the antioxidant activity of the phenolics is “encapsulated” or “protected” by the proteins. In contrast, LPE–2 extracted more carbohydrates, which may have interfered with detecting phenolic compounds in the TPC analysis. This interference could falsely elevate TPC values, masking the actual phenolic content and affecting the accuracy of antioxidant activity assessments.

As expected, a high TPC (Figure 3C) was observed in the extracts obtained through the extraction performed with ethanol/water (70:30 *v*/*v*), LPE–2 (12.69 g GAE 100 g^−1^), and PLE (14.02 GAE 100 g^−1^). This result indicates that the second low-pressure stage (LPE–1) was less efficient than PLE for extracting phenolic compounds. This step also achieved considerable flavonoid recovery, with high values for the PLE extract (0.52 g QE 100 g^−1^).

This step also allowed for extracts with significantly higher antioxidant capacity by ABTS (LPE–2: 4295.59 and PLE: 3051.37 µmol TE g^−1^, both statistically equal) than obtained in the first biorefinery step (*p*-value < 0.05) (Figure 3D). This pattern shows that solvent polarity, particularly the use of ethanol/water mixtures, was a determinant for antioxidant recovery, surpassing the efficacy of CPME. The composition and subsequent biological activity of propolis are significantly influenced by various factors, such as geographic origin, cultivation practices, and extraction methodologies [32]. This inherent variability often complicates direct result comparisons across studies. Our findings, demonstrating a higher ABTS value than DPPH, align with Ferreira et al. [33], who reported a pronounced ABTS antioxidant capacity (1050 µmol TE g^−1^) compared to DPPH (750 µmol TE g^−1^) in extracts acquired from Brazilian green propolis produced via seven-day maceration in a 70:30 ethanol/water solution at room temperature. In addition, Santana Neto et al. [34] also reported an ABTS value (1945 µmol TE g^−1^) higher than the DPPH value (741 µmol TE g^−1^) for Brazilian green propolis extract acquired using ethanol (100%) by ultrasound extraction (40 kHz). As previously reported, those results underscore that the antioxidant compounds in propolis extracts encompass both polar and non-polar molecules.

Corroborating the first extraction step, the LPE–2 extract exhibited superior AChE and BChE inhibition compared to PLE. The LPE–2 extract (AChE: 163.92 and BChE: 173.97 μg mL^−1^) demonstrated a stronger enzymatic inhibition than PLE (AChE: 349.36 and BChE: 485.11 μg mL^−1^), as depicted in Figure 3E. The neuroprotectivity activity of the second biorefinery step was intermediated between the first and the third steps. Among the sequential extraction steps, the LPE–2 extract demonstrated the highest LOX inhibitory activity, with a value of 74.40 mg QE g^−1^, significantly surpassing that of the other extracts. The literature primarily attributes propolis’ anti-inflammatory effects to its phenolic and flavonoid composition, notably flavanols (quercetin, rutin, and morin) and flavanones (hesperetin and hesperidin) [22]. The high total phenolic and flavonoid contents observed in LPE–2 and PLE extracts (Figure 3C) correlate with their superior anti-inflammatory capacities. This strong LOX inhibition suggests that the ethanol/water solvent mixture employed in LPE–2 may have extracted a unique combination of anti-inflammatory bioactive compounds, potentially including flavonoids and terpenoids, which are known for their ability to inhibit inflammatory enzymes. Phenolic and flavanols exhibit multiple anti-inflammatory mechanisms, including potent antioxidant action, the regulation of inflammatory cells, the inhibition of enzymes involved in arachidonic acid metabolism (phospholipase A2, LOX, and nitric oxide synthase), control of pro-inflammatory cytokine and mediator production, and the suppression of pro-inflammatory gene expression [4]. This makes the LPE–2 fraction highly promising for anti-inflammatory applications, especially in pharmaceuticals targeting chronic inflammation and pain relief.

#### 2.2.3. Third Biorefinery Step: SWE and LPE–3

As previously reported for the second step, since a wide range of compounds were extracted in the first and second steps, a lower yield was achieved for the third step, in which SWE (31.85%) presented a high process yield compared with LPE–3 (11.99%). The low yield at this stage reflects the reduced carbohydrate and protein contents, TPC, and TFC in the residual material from the two first steps. Among all extracts, SWE exhibited the lowest total carbohydrate content at 0.51 g Glu 100 g^−1^. Regarding protein content, LPE–3 and SWE showed moderate levels, with values of 0.96 mg BSA g^−1^ and 1.88 g BSA 100 g^−1^ (Figure 3B), respectively. The carbohydrate and protein contents were mostly extracted during the PLE and LPE–2 stages, resulting in a depleted matrix of these components.

The HPE process (Figure 3C) had a SWE yield of 2.82 g GAE 100 g^−1^ for TPC and 0.12 g QE 100 g^−1^ for TFC. A low TFC value was recovered for LPE–3 (0.04 g QE 100 g^−1^). The overall flavonoid yield followed the pattern PLE > SFE > SWE (similar behavior to TPC) for HPE and LPE–1 > LPE–2 > LPE–3 for LPE. Solvent polarity and process conditions significantly influenced phenolic and flavonoid extraction. Since a wide range of TPC and TFC was extracted in the first (LPE–1 and SFE) and second (LPE–2 and PLE) steps, only the remaining and less accessible portion of phenolic and flavanol compounds was recovered in the subsequent extractions (LPE–3 and SWE, both with statistically equal values).

The third biorefinery step provided the best AChE (79 μg mL^−1^) and BChE (118 μg mL^−1^) inhibitory activities for the LPE–3 process, while SWE exhibited the lowest inhibitory activity among all HPE extracts (AChE: 590 and BChE: 534 μg mL^−1^). These results indicated that the inhibition of AChE and BChE by propolis extracts may not correlate with their antioxidant properties or total phenolic content, since a higher TPC and antioxidant capacity by ABTS and DPPH were observed for extracts acquired from the first and second biorefinery steps. Instead, it implies that different compounds of propolis extracts are responsible for the observed AChE and BChE inhibitory effects. Beyond phytochemicals, other natural substances such as peptides [35] and polysaccharides [36], both observed in SWE and LPE–3 extracts, may also contribute to enzyme inhibition, highlighting the complex composition of propolis, which includes various compounds beyond those characterized in this study. Galantamine, a standard for cholinesterase inhibition, shows very low IC_50_ values for AChE (0.85 μg mL^−1^) and BChE (2.85 μg mL^−1^), demonstrating its high potency. Despite this, propolis extracts also exhibit significant inhibitory effects on AChE and BChE, suggesting that they offer promising natural alternatives with considerable potential for cholinesterase inhibition. Moderate LOX inhibitory activity was achieved for the third extract (33.88 mg QE g^−1^ for SWE and 33.32 mg QE g^−1^ for LPE–3) with no statistical differences.

#### 2.2.4. Global Process Evaluation

The global yield data (Figure 3A) reveal that the sequential HPE achieves the highest overall yield at 80.86%, nearly 10% higher than the LPE (71.43%), highlighting the superior performance of high-pressure methods. In green propolis extraction, the LPE method (LPE–1 + LPE–2 + LPE–3) yielded 6.19 g BSA and 12.82 g Glu 100 g^−1^, while HPE (SFE + PLE + SWE) recovered 13.60 g BSA and only 4.05 g Glu 100 g^−1^ of protein and carbohydrate, respectively. This indicates that HPE extracts phenolics more effectively, while minimizing carbohydrates, leading to more concentrated and purer extracts. This enhances extract quality, reduces the need for further purification, and promotes environmental sustainability. Nevertheless, the environmental impact of both processes will be compared in the next section. High-purity phenolic extracts are preferable for applications in pharmaceuticals, nutraceuticals, and cosmetics, as sugars can diminish phenolic potency, reduce stability, and promote microbial growth, which accelerates product degradation [37].

In conclusion, the sequential extraction process applied to green propolis yielded three distinct extracts:

- SFE is enriched in lipids, lipophilic compounds, phenolics, terpenes, and waxes, making it ideal for cosmetics and skincare. It can also serve as an emollient and moisturizer, provide antioxidant and anti-inflammatory protection, offer fragrance and therapeutic effects, thicken products, and form a protective layer on the skin [1,38,39];
- PLE is abundant in phenolic compounds, flavonoids, carbohydrates, and proteins. It has potential uses in the food industry as a natural antioxidant and preservative, in cosmetics for anti-aging and moisturizing, in nutraceuticals for functional supplements, and in pharmaceuticals for preventive therapies. It can also be used in bioactive packaging to extend the shelf life of perishable products [1,10,38];
- SWE contains proteins and other less accessible compounds with moderate anti-inflammatory activity. It is suitable for pharmaceuticals as a topical treatment or supplement, for cosmetics to soothe and reduce redness in skincare products, and for the food industry and nutraceuticals as a source of proteins and bioactive compounds. Additionally, it has potential in biomedical applications, including tissue regeneration and therapeutic agents [11,21,39].

### 2.3. Greenness Assessment of the HPE and LPE Processes

The greenness of the sequential high- and low-pressure extraction processes for recovering high-added-value compounds from green propolis was evaluated and compared by employing the *Path2Green* software [15]. The greenness evaluation is based on 12 principles, broadly discussed below.

Principle 1 (weight 6) is to do with the selection of biomass. Propolis, a bee-produced resin derived from plant materials, is the primary raw material for extraction. Given its natural origin, propolis collection is generally considered to have minimal environmental impact. Based on this assessment, a score of 0.75 (for both processes, LPE and HPE) was assigned to this principle.

Transportation distance is a primary factor influencing the environmental impact of transporting propolis (Principle 2, weight 5). Considering transport mode, vehicle efficiency, and packaging, a score of 50 km was assigned to the considered transportation scenario, which involved medium distances, road transport, efficient vehicles, and sustainable packaging.

Principle 3 (weight 2.5) involves pre-treatment, which is crucial for effective propolis extraction, but should be minimized to lower costs and environmental impact. Grinding, a physical pre-treatment used in this work, improves particle size reduction and compound accessibility, aiding solvent penetration and analyte solubilization, thus increasing yield [40]. This pre-treatment method has a low environmental impact score of −0.20 for both sequential processes.

Following biomass pre-treatment, the extraction process started with solvent selection (Principle 4, weight 6). This study employed bio-based solvents, including CPME, ethanol, and water, which align with the CHEM21 guidelines for sustainable solvents [15]. As both (LPE and HPE) processes operated the same solvent system, a maximum score of +1 was assigned for both.

Principle 5 (weight 5) outlines the advantages of HPE over LPE in terms of scalability. HPE operates semi-continuously and in flow, enhancing scalability by allowing uninterrupted extraction. This method increases the consistency of production and ease of scaling for large operations, earning it a score of +1. On the other hand, LPE’s batch-mode operation, while suitable for smaller production scales, presents economic and operational limitations when scaling up. The requirement for manual intervention and downtime between cycles can result in higher labor costs and lower productivity, making it less economically viable for large-scale applications compared to the continuous nature of HPE. Consequently, LPE extraction receives a score of −1 for its batch nature, which limits its scalability.

Purification is essential for achieving high-purity extracts (Principle 6, weight 2.5). While the direct application of crude propolis extract is possible in certain cases, specific applications often require post-treatment to isolate compounds or enhance purity. HPE extracts require less extensive purification due to their initial purity, while LPE extracts typically necessitate more rigorous processes. However, considering the minimal purification required for HPE and LPE processes when employing solvent-free methods like membrane filtration, a score of +0.50 was assigned to both.

Principle 7 (weight 4) focuses on maximizing biomass utilization and yield. The LPE process is characterized by its semi-exhaustive nature. It extracts a lower portion of target compounds from the biomass, as observed in the lower global yield, than HPE (Figure 3A). This lower yield equates to weak biomass utilization, resulting in a score of −0.5. On the other hand, HPE is seen as an exhaustive extraction method, successfully maximizing the extraction and recovery of target compounds. The higher overall yield achieved with HPE reflects a more efficient and sustainable use of biomass, leading to reduced waste and better resource utilization. Therefore, HPE is given a neutral score of 0, due to its effectiveness in extracting the maximum possible yield from the biomass.

Principle 8 (weight 2.5) optimizes post-treatment processes to enhance extract quality. Complete solvent removal is crucial for extracts intended for food, cosmetic, or nutraceutical applications, to ensure product safety and quality. Sustainable purification methods, such as adsorption, chromatography, and membrane filtration, should prioritize green solvents, minimize resource consumption, and reduce waste. Efforts to recycle and reuse materials enhance the overall sustainability of the process (Martínez-Pérez-Cejuela and Gionfriddo, 2024). HPE and LPE received a score of 0.5 due to the possibility of incorporating these post-treatment practices into the possible industrial extraction processing of propolis.

Principle 9 (weight 4) addresses energy use and sources. The sustainability score is notably reduced when non-renewable energy sources are used, which is common in industrial settings. Due to the reliance on non-renewable energy, both methods receive a low sustainability score of –1. However, a study by Irakli et al. [41] showed that HPE is more energy efficient and produces lower CO_2_ emissions than LPE (represented by Soxhlet). HPE consumes 0.80 KWh g^−1^ of the extract, significantly lower than the 7.78 KWh g^−1^ consumed by LPE. Regarding CO_2_ emissions, HPE generates 0.64 kg of CO_2_ per gram of extract, much lower than the 6.21 kg of CO_2_ emitted by Soxhlet. These data demonstrate that HPE is more energy efficient and has a much lower environmental impact, making it a more sustainable alternative to LPE. This principle highlights the importance of choosing energy sources to mitigate the environmental impact of extraction processes.

Principle 10 (weight 4.5) emphasizes safety and applicability across various domains. While CPME, a bio-based solvent, offers advantages in extraction, its use is currently restricted to food applications. This limitation narrows the potential market for CPME-based extracts to non-food sectors such as cosmetics, biochemistry and genetics, chemistry, microbiology, dermatology, veterinary, and disinfection [38]. The versatility of the extracts, especially those obtained using HPE, is evident in their potential applications in diverse fields such as cosmetics, pharmaceuticals, and nutraceuticals. This versatility underscores the importance of the extraction process in producing multifunctional bioactive compounds and justifies a high sustainability score of 0.83. Further optimization could expand the range of applications, thereby increasing the value and market reach of these extracts.

Industrial extraction processes must prioritize closed-loop systems and material reuse to align with sustainability principles (Principle 11, weight 6). Solvent recovery and recycling, as well as waste minimization, are crucial. The implementation of solvent recovery offers a valuable opportunity to lower production costs, enhance the environmental sustainability of the process, and decrease emissions and other environmental impacts typically linked to industrial operations [42]. Although both LPE and HPE used bio-based solvents (except hexane in LPE), their recovery efficiencies differed significantly. HPE’s closed-loop system enables over 80% recovery versus 71% for LPE, minimizing waste and resource consumption, impacting its overall sustainability score.

Effective waste management is crucial for sustainable extraction (Principle 12, weight 6). Optimizing extraction yields, reusing resources, and reducing waste generation are essential. The E-factor, quantifying waste relative to product output, evaluates process efficiency. Lower E-factors signify better sustainability. Given process efficiencies (PLE: 71.43% and HPE: 80.86%) and calculated E-factors (LPE: 39.99 and HPE: 26.67), HPE (score: 0.46) demonstrates superior waste management practices compared to LPE (score: 0.2).

**Figure 5:**
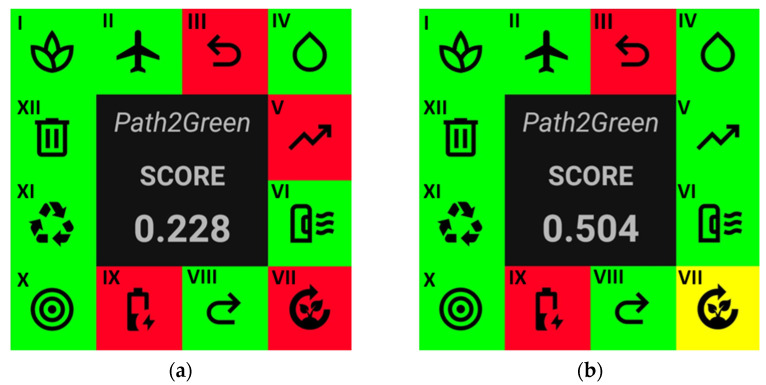
Comparative environmental impact assessment of (**a**) LPE and (**b**) HPE biomass processing: analysis of biomass (I), transport (II), pre-treatment (III), solvent (IV), scale-up (V), purification (VI), yield (VII), post-treatment (VIII), energy (IX), application (X), repurposing (XI), and waste (XII). Indications of a good score (green), areas requiring attention (yellow), and poor score (red).

In conclusion, the scores for LPE and HPE were 0.228 and 0.504, respectively (Figure 5). While both methods employ sustainable practices, HPE stands out for its scalability, purification efficiency, and waste management, as evidenced by its lower E-factors and optimized resource use. LPE, although effective, faces challenges in scalability and generates more waste. Overall, the greenness assessment underscores that HPE aligns more closely with sustainable development goals and green chemistry principles, particularly in its waste reduction, recovery efficiency, and energy optimization. By integrating HPE into industrial biorefinery setups, companies can not only meet environmental regulations, but also contribute to broader global sustainability initiatives, such as reducing greenhouse gas emissions and promoting responsible resource usage. HPE better aligns with sustainable extraction principles, making it the preferred choice for environmentally conscious processes.

## 3. Material and Methods

### 3.1. Propolis Biomass and Materials

Green propolis from *Baccharis dracunculifolia* was purchased from Breyer^®^ (Formigas—Minas Gerais, Brazil), containing 6.55% moisture, 18.93% oil, 3.23% carbohydrates, 11.87% protein, 3.89% ashes, and 55.55% of other compounds, such as fiber, resins, balsams, phenolic acids, flavonoids, and aromatic compounds. The detailed material used in this work is described in Supplementary Material S.1.

### 3.2. Biorefining Green Propolis: Sequential High-Pressure Extraction Process

A sequential high-pressure process driven by the three-step biorefinery concept was designed to obtain different green propolis extracts with distinctive functionalities. Figure 4 illustrates the extraction conditions (solvent, flow rates, pressures, temperatures, and time) applied to each stage. High-pressure extractions (HPEs) were conducted using a custom-built high-pressure extractor with high-pressure CO_2_ and a co-solvent pump (PU-2080Plus, Jasco, Japan) in a semi-continuous flow setup. A manual metering valve (Swagelok, Solon, OH, USA) controlled the extraction pressure.

In addition to evaluating the high-pressure process, the biorefinery was also studied at low pressure, using a Soxhlet apparatus at the same solvent/feed ratio (S/F) and extraction time (Figure 4) (low pressure extraction (LPE)).

In the cases of both HPE and LPE, the solvent polarity of the different steps was chosen in ascending order, starting from low polarity solvents and ending with water as the extraction solvent.

#### 3.2.1. Supercritical Fluid Extraction (SFE)

The first step of the biorefinery was optimized using a full factorial design (FFD) to extract the non-polar fraction of propolis. The independent evaluated variables were (i) temperature (40, 50, and 60 °C), selected to prevent the degradation of thermolabile compounds and to facilitate the solubilization of propolis non-polar components (waxes), which have a melting point between 60 and 70 °C [43] and (ii) co-solvent cyclopentyl methyl ether (CPME) proportion (10, 20, and 30%), a bio-based and environmentally friendly solvent with intermediate polarity (4.7) [44], compared to ethanol (5.2) [44], enabling a broader extraction range, including waxes [45].

FFD accounted for 9 randomized runs, all performed in duplicate (Table S1). For each SFE, 1 g of ground green propolis and 20 g of glass beads (dispersing agent) were placed in the stainless steel extraction cell of 25 mL. The cell was fitted with glass wool and cellulose filters (Restek, Bellefonte, PA, USA) at both ends on the side of the solvent inlet (bottom) to avoid carrying solid particles with the extract. The flow rate and the pressure were maintained constant, 4 mL min^−1^ and 20 MPa, respectively, during SFEs. The pressure (200 bar) was selected based on the study by Alaydi et al. [46], which was optimal for the temperature range of 50–70 °C. A high solvent flow rate (4 mL min^−1^) ensured efficient extraction and prevented solvent saturation.

A preliminary kinetic study of 60 min was performed to establish the extraction time under the central conditions of the experimental design (20% CPME and 50 °C). Fractions were collected every 10 min. After each extraction, CPME was evaporated using N2 (TurboVap LV—Caliper, Biotage AB, Upsala, Sweden) at room temperature to determine the global extraction yield. Dried extracts were stored at −20 °C until characterization assays.

The responses of the experimental design were total phenolic content (mg GAE g^−1^), antioxidant capacity (ABTS and DPPH, µmol TE g^−1)^, and anti-inflammatory capacity (LOX, mg Quercetin equivalent, QE g^−1^), according to Section 2.3. The effect of temperature and CPME ratio on extraction was also evaluated on the profile of non-polar compounds of the extracts (fatty acids, phenolics, terpenoids, and sterols), determined by gas chromatography associated with mass detection (GC–MS). The results obtained in this first stage were compared to those obtained by a conventional Soxhlet extraction using hexane, an S/F of 50, for 6 h. The SFE-optimized conditions of co-solvent percentage and temperature were employed in the biorefinery process (Figure 4), and the extract was evaluated for yield (%), TPC, TFC, antioxidant capacity (ABTS and DPPH), carbohydrate and protein contents, neurodegenerative effects (AChE and BChE inhibitory activity), and anti-inflammatory potential (LOX inhibition).

After selecting the optimum temperature and solvent flow, HPE was carried out, starting with SFE, as shown in Figure 1. SFE, PLE, and SWE were performed sequentially on the same equipment. After each extraction, CO_2_ was pumped through the extraction cell to remove residual solvent and dry the raw material before proceeding onto the next extraction.

#### 3.2.2. Pressurized Liquid Extraction (PLE)

The residue from the SFE was used as a raw material for PLE, focusing on obtaining a fraction rich in phenolic compounds. The fixed PLE conditions employed (ethanol: water (70:30 *v*/*v*), flow of 2 mL min^−1^, S/F of 60, 130 °C, and 100 bar for 15 min) were defined according to the preliminary results of a study by the research group. This extraction was performed in duplicate, and the obtained extracts were analyzed using the same analytical protocol employed to characterize the optimized SFE extract (see Section 3.2.1).

#### 3.2.3. Subcritical Water Extraction (SWE)

SWE using optimized process parameters from Shin et al.’s [11] study (flow of 2 mL min^−1^, S/F of 133.33, 180 °C, and 100 bar for 20 min) was carried out to extract protein, sugars, and other less accessible compounds still present in the PLE residue. SWE was performed in duplicate, and the obtained extracts were analyzed using the same analytical protocol employed to characterize the optimized SFE and PLE extracts (see Section 2.2.1 and Section 2.2.2).

#### 3.2.4. Sequential Low-Pressure Extraction (LPE) Process

The sequential low-pressure extractions (LPEs) were performed in a Soxhlet apparatus using similar conditions employed in high-pressure biorefinery. In the first step, CPME was used as a solvent to compare the extraction efficiency, quality, and chemical composition with SFE extracts (Section 2.2.1). After the first extraction (LPE1), the obtained propolis residue was dried and ground (50 °C for 24 h) before being subjected to the second extraction (LPE2). The same procedure was followed for the third extraction (LPE–3). Sequential extractions used the same solvents as HPE: EtOH/H_2_O (70:30 *v*/*v*) (LPE–2) and water (LPE–3), with the same solvent-to-feed mass ratios (120, 60, and 133.3) and extraction times (30, 15, and 20 min, respectively). After LPEs, CPME and ethanol were evaporated using N_2_ (TurboVap LV—Caliper, Biotage AB, Upsala, Sweden), and water was removed by lyophilization to determine the global extraction yield. The extracts were kept at −20 °C. LPEs were performed in duplicate, and the obtained extracts were analyzed using the same analytical protocol employed to characterize the biorefinery approach for HPE (optimized SFE, PLE, and SWE extracts; see Section 3.2.1, Section 3.2.2 and Section 3.2.3).

### 3.3. Extracts Characterization

#### 3.3.1. Global Extraction Yield (%)

The extraction yield was calculated according to the Supplementary Material S.2.1.

#### 3.3.2. Total Phenolic Content (TPC)

TPC was estimated using the Folin–Ciocalteu assay, following the colorimetric method described by [47]. The detailed protocol is described in Supplementary Material S.2.2.

#### 3.3.3. Total Flavonoid Content (TFC)

TFC was quantified using the spectrophotometric method proposed by Dowd (1959) [1,48] and Mammen and Daniel (2012) [49]. The detailed protocol is provided in Supplementary Material S.2.3.

#### 3.3.4. Biological Activities

The extracts’ in vitro activities were assessed for antioxidant capacity using ABTS-2,2′-azino-bis-(3-ethylbenzothiazoline-6-ammonium sulfonate) [50] and DPPH-2,2-diphenyl-1-picrylhydrazyl [51] assays. Additionally, the extracts were evaluated for neuroprotective effects, AChE and BChE inhibition [28], and their anti-inflammatory potential via lipoxygenase (LOX) inhibition [52]. A detailed description of these methods is available in Supplementary Material S.2.4.

#### 3.3.5. Total Carbohydrate and Protein Contents

The total carbohydrate content of the extracts was determined through the Phenol-sulfuric acid method [53] and the total protein content of the extracts was evaluated using the Bradford method, as detailed in Supplementary Material S.2.5.

#### 3.3.6. Chemical Characterization by Gas Chromatography–Mass Spectrometry

GC–MS was carried out to determine the chemical composition of the extracts. The methods used for these analyses are described in Supplementary Material S.2.6.

### 3.4. Greenness Assessment of HPE and LPE Processes

*Path2green* software was employed to compare the greenness metrics of the HPE with LPE [15]. The greenness metrics for HPE and LPE were assessed based on the 12 principles of green chemistry: biomass (principle 1), transport (principle 2), raw material pre-treatment (principle 3), solvent (principle 4), scalability (principle 5), purification (principle 6), yield (principle 7) post-treatment (principle 8), energy consumption (principle 9), application (principle 10), repurposing (principle 11), and waste management (principle 12). The results were illustrated as a pictogram, presenting a comprehensive score.

### 3.5. Statistical Analysis

The results were expressed as the average, followed by the standard deviation. One-way analysis of variance (ANOVA) was conducted to determine significant differences between treatments (*p* < 0.05). All statistical analyses were evaluated using Minitab 17 software (Minitab, LLC, State College, PA, USA). For the FFD, the effect of each experimental parameter on the response variables was studied at a 95% confidence level.

## 4. Conclusions

This study shows that the sequential high-pressure extraction (HPE) method is a sustainable and efficient approach for biorefining Brazilian green propolis, outperforming traditional low-pressure extraction (LPE) with an overall yield of 80.86%. HPE produced three distinct extracts: SFE concentrated lipids and lipophilic compounds, including fatty acids, terpenoids (2,6-di-tert-butyl phenol), phenolics (benzoic acid), and sterols (ergost-25-ene-3,5,6,12-tetrol) as the main compounds; PLE was rich in phenolics, proteins, flavonoids, and carbohydrates; and SWE extracted proteins and less accessible compounds. Although HPE selectively targeted specific compounds, LPE extracts demonstrated superior inhibition of AChE, BChE, and LOX, suggesting that a symbiosis of compounds enhances neuroprotective and anti-inflammatory effects. Phenolics, known for their antioxidant properties, can help reduce oxidative stress, while flavonoids may modulate enzyme activity and inflammatory pathways. Together, these compounds work synergistically, amplifying each other’s effects and enhancing the overall bioactivity of LPE extracts. Environmentally, HPE is more eco-friendly, reducing solvent usage by 25% and generating less waste through a closed-loop solvent recovery system. This efficient resource management results in a 40% reduction in overall environmental impact compared to LPE, aligning with key green chemistry principles such as waste prevention and resource efficiency. In conclusion, this study identifies high-pressure extraction (HPE) as a sustainable biorefining strategy for propolis, providing benefits in yield, environmental impact, and industrial applicability. HPE promotes the integration of green technologies, supporting sustainability goals and advancing green industries. Despite the promising results, the economic viability of the process is crucial for industrial applications and could be explored in future research.

## Figures and Tables

**Table 1:** Predicted and experimental values of the responses at the optimum conditions (78,9% CO_2_, 21.1% CPME, 20 MPa, and 60 °C).

Responses	Predicted Values			Experimental Values
	−95%	Optimum Values	+95%	
**Yield (%)**	51.0	56.7	62.4	* 55.9 ± 0.1
**TPC (mg GAE g^−1^)**	147.0	166.7	186.4	149 ± 5
**ABTS (µmol TE g^−1^)**	2068.0	2496.0	2924.0	2455 ± 333
**DPPH (µmol TE g^−1^)**	598.2	634.7	671.3	620.0 ± 0.6
**LOX (mg QE g^−1^)**	14.9	21.5	28.0	15.2 ± 0.6

* Experimental data are expressed as the mean ± SD.

## Data Availability

The data that support the findings of this study are available from the corresponding author upon reasonable request.

## Supplementary Materials

The following supporting information can be downloaded at: https://www.mdpi.com/article/10.3390/molecules30010189/s1, S.1. Materials, S.2. Extracts characterization; Figure S1: SFE kinetic of green propolis at 4 mL min^−1^, 200 bar, 50 °C, and 20% CPME as cosolvent; Figure S2: Pareto chart of standardized effects (a) yield, (b) TPC, (c) ABTS, (d) DPPH, and (e) LOX. Factors (A) co-solvent, (B) temperature. α = 0.05; Table S1: Experimental design for the full-factorial design (FFD) applied to obtain the optimum process parameters (co-solvent—CPME and temperature) of the SFE of bioactive molecules from green propolis as first step of biorefinery process. Extraction pressure 20 MPa and flow 4 mL min^−1^ constants in all experiments; Table S2: ANOVA of the fitted. Full Factorial model for SFE yield (%) from green propolis; Table S3: ANOVA of the fitted Full Factorial model for TPC (mg GAE g^−1^) from green propolis; Table S4. ANOVA of the fitted Full Factorial model for ABTS (µMol TE g^−1^) from green propolis; Table S5: ANOVA of the fitted Full Factorial model for DPPH (µMol TE g^−1^) from green propolis; Table S6: ANOVA of the fitted Full Factorial model for LOX (mg QE g^−1^) from green propolis; Table S7. Tentatively identified compounds by GC–MS/MS analysis from SFE extracts of green propolis extracts.
