# Supplementary material for: Biorefining Brazilian Green Propolis: An Eco-Friendly Approach Based on a Sequential High-Pressure Extraction for Recovering High-Added-Value Compounds
Source: Molecules. 2025 Jan 6;30(1):189. doi: 10.3390/molecules30010189 (PMC11722997; doi:10.3390/molecules30010189)
Supplement: Supplementary file 1 [file molecules-30-00189-s001.zip › molecules-3402294-supplementary.pdf]

molecules

MDPI

*Supplementary Materials*

# Biorefining Brazilian green propolis: An eco-friendly approach based on a sequential high-pressure extraction for recovering high-added-value compounds

**Guilherme Dallarmi Sorita [#,1,2,*], Wilson Daniel Caicedo Chacon [#,1,2], Monique Martins Strieder [1], Camilo Rodriguez-García [1], Alcilene Monteiro Fritz [1,2], Silvani Verruck [1,3], Germán Ayala Valencia [1,2] and José A. Mendiola [1,*]**

[1] Foodomics Laboratory, Institute of Food Science Research (CIAL) (CSIC-UAM), Nicolás Cabrera 9, 28049 Madrid, Spain; guilhermedallarmi@hotmail.com; w.caicedo.ch@gmail.com, monique_strieder@hotmail.com; crodriguezga@unal.edu.co; silvaniverruck@gmail.com; gayalavalencia@gmail.com; alcilenemonteiro@gmail.com; j.mendiola@csic.es

[2] Department of Chemical and Food Engineering, Federal University of Santa Catarina, Florianópolis, SC, 88040-900 Brazil

Department of Food Science and Technology, Federal University of Santa Catarina, Rodovia Admar Gonzaga, 1346, Itacorubi, Florianópolis 88034-000, SC, Brazil

[#] Both authors contributed equally

* Correspondence: j.mendiola@csic.es and guilhermedallarmi@hotmail.com

## S.1. Materials

Cyclopentyl methyl ether – CPME and Folin-Ciocalteu reagent was purchased from VWR Chemicals (France), Gallic acid from AlfaAesar (99%, Kandel, Germany), and Na2CO3 from Labkem (99%, Barcelona, Spain). For ABTS (2,2′-azino-bis-3-ethylbenzthiazoline-6-sulphonic acid) assay, potassium persulfate (K2S2O8) was acquired from Montplet & Estaban SA (98%, Madrid, Spain), ABTS and potassium dihydrogen phosphate (K2HPO4) from Sigma Aldrich (> 98%, Steinheim, Germany), and sodium hydrogen phosphate anhydrous (Na2HPO4) from Merck (Darmstadt, Germany). DPPH (1,1-diphenyl-2-picrylhydrazyl) was purchased from TCI Chemicals (Tokyo, Japan, 97%). Trolox, used as the standard reference for ABTS and DPPH methods, was purchased from Sigma Aldrich (98%, Steinheim, Germany). For reducing sugar quantification, 3,5-Dinitrosalicylic acid (DNS) and sodium potassium tartrate were obtained from Sigma (St Louis, MO, USA) and NaOH from Merck Millipore (Darmstadt, Germany, 99%). For the neuroprotective and anti-inflammatory assays, phosphatidylcholine acetylcholinesterase (AChE) Type V1-S from Electrophorus electricus, butyrylcholinesterase (BChE) from equine serum, acetylthiocholine iodide (ACth), butyrylcholine iodide (BCth), Trizma base (2-amino-2-(hydroxymethyl)-1,3-propanediol) (>99.9 %), Fluorescein sodium salt, and quercetin were obtained from Sigma-Aldrich (Madrid, Spain). Lipoxidase from Glycine max, 4-(amino-359 sulfonyl)-7-fluoro-2,1,3-benzoxadiazole (ABD-F), and galantamine hydrobromide were purchased from TCI Chemicals (Tokyo, Japan). Ultrapure MiliQ water was obtained from a Milipak® Express 40 system (Merk-Milipore, Darmstadt, Germany). N-Methyl-N-(trimethylsilyl)trifluoroacetamide (MSTFA) for sample derivatization for GC-MS was purchased from Fluka Analytical.

## S.2. Extracts characterization

### *S.2.1. Global extraction yield (%)*

The extraction yield was calculated as a percentage (%) of the mass of extract ($m_E$) relative to the total mass of raw material ($m_{RW}$), according to Equation 1.

$$\text{Yield (\%)} = \left(\frac{m_E}{m_{RW}}\right) * 100 \quad (1)$$

*S.2.2. Total phenolic content (TPC)*

TPC was estimated using the Folin-Ciocalteu assay, following the colorimetric method described by (Kosar et al., 2005). Briefly, 10 µL of the sample (diluted in ethanol) was mixed with 600 µL of water, and then 50 µL of Folin-Ciocalteu reagent was added. After one minute, 150 µL of 20% (w/v) Na2CO3 was added, and the volume was adjusted to 1 mL (90 µL) with water. The solutions were then agitated and stored in the dark for 2 h at ambient temperature (25 °C). After that, the absorbance was read at 760 nm (BioTeck Synergy HT microplate reader, Winooski, Vermont, USA). Gallic acid (0.03 to 2 mg $mL^{-1}$) was utilized to construct a standard curve ($R^2$ = 0.99). TPC results were expressed as mg of Gallic acid equivalent (GAE) $g^{-1}$ of extract ± standard deviation from triplicate measurements.

*S.2.3. Total Flavonoid Content (TFC)*

TFC was quantified using the spectrophotometric method proposed by Dowd (1959) and Mammen & Daniel (2012). First, 100 µL of the sample was mixed with 140 µL of the respective extraction solvent and 60 µL of 8 mM aluminum chloride. The mixture was incubated for 30 min in the dark, and absorbance was measured at 425 nm (BioTeck Synergy HT microplate reader). The sample absorbances were corroborated with a Quercetin analytical curve ($R^2$ = 0.99). Results were expressed as mg QE $g^{-1}$ of extract ± standard deviation from triplicate measurements.

*S.2.4. In vitro antioxidant, neuroprotective, and anti-inflammatory activity*

ABTS method: Antioxidant capacity was measured using the ABTS method according to the methodology proposed by Re et al. (1999). First, ABTS+• radical cation was generated by combining a 7 mM ABTS solution with a 2.45 mM potassium persulfate solution without light at room temperature (25 °C) for 16 h. Then, the ABTS+ solution was diluted in distilled water until it reached an absorbance of 0.7 (± 0.05) at 734 nm. Finally, 20 µL of diluted extracts were mixed with 280 µL of ABTS+• solution and incubated in the dark for 30 min, followed by absorbance measurement (BioTeck Synergy HT microplate reader, Winooski, Vermont, USA) at 734 nm. Trolox was used as the standard reference curve ($R^2$ = 0.99). Results were described as µmol Trolox Equivalent per gram of extract (µmol TE $g^{-1}$ extract) ± standard deviation from triplicate measurements.

DPPH method: DPPH assay was also employed to assess the antioxidant capacity of the propolis extracts (Brand-Williams et al., 1995). Shortly, 10 µL of each extract (dissolved in ethanol) was combined with 290 µL of DPPH ethanolic solution (0.6 µmol $L^{-1}$). Following a 30-min reaction at room temperature and without light, absorbance was measured at 517 nm using a BioTeck Synergy HT microplate reader (Winooski, Vermont, USA). Trolox was also utilized to construct a reference curve ($R^2$ = 0.99). The results were expressed in µmol TE $g^{-1}$ extract ± standard deviation by triplicate measurements.

Acetylcholinesterase (AChE) and butyrylcholinesterase (BChE) inhibition: Before evaluating the extract's capacity, the substrate concentration (ACth or BCth) was adjusted to ensure the enzyme operated at half its maximum velocity based on the Michaelis-Menten constant (Km). Inhibitory capacity was measured in a 96-well plate by adding 100 µL of extract sample (at different concentrations), 100 µL of Tris-HCl buffer (pH 8), and 25 µL of AChE or BChE (0.8 U $mL^{-1}$ in buffer) added in each plate well and then incubated for 10 min. Then, the plate was placed inside a microplate reader (Biotek, Winooski, VT, USA) with an auto dispenser coupled, which introduced 25 µL of ABD-F (125 µM in the buffer) and 50 µL of ACth (at a concentration calculated by KM in ultrapure water) to each well. Finally, the reactive medium was agitated, and the absorbance was measured at intervals of 10 s for 15 min at 37 °C at 389 nm (λexcitation) and 513 nm (λemission). Galantamine was used as a positive control. Measurements were carried out in triplicate, and the results were expressed as IC50 (the extract concentration required to reduce 50% inhibition of enzyme activity).

LOX Inhibitory Activity: The LOX inhibition capacity, as measurement of anti-inflammatory activity, was evaluated using a fluorescence assay based on enzymatic kinetics described by Whent et al. (2010). Briefly, 100 µL of extract (2.5 to 10 mg $mL^{-1}$), 75 µL of fluorescein (1 µM) in 150 mM Tris/HCl buffer (pH 9), 60 µL of LOX (208 U $\mu L^{-1}$ in buffer), and 100 µL of linoleic acid (substrate, with concentration adjusted based on the Km value in EtOH) were added to each well. The mixture was placed in a microplate reader (Biotek, Winooski, VT, USA), and fluorescence kinetic measurements were recorded at 485 nm (λexcitation) and 530 nm (λemission) every

minute for 15 min at 25 °C. Quercetin was used to corroborate the sample's results. Measurements were performed in triplicate, and results were expressed as mg QE $g^{-1}$ of extract.

*S.2.5. Total carbohydrate and protein content*

The total carbohydrate content of the extracts was determined through the Phenol-sulfuric acid method (Masuko et al., 2005). Briefly, 100 µL of each extract, 300 µL of concentrated $H_2SO_4$, and 90 µL of phenol (5 % w/v) were mixed. This mixture was heated to 95 °C for 5 min (Eppendorf® Thermomixer Comfort, Hamburg, Germany) and cooled down rapidly by transferring to a water bath. The absorbance of the samples was measured at 540 nm (BioTeck Synergy HT microplate reader, Winooski, Vermont, USA). Water was used as blank. The sample's absorbances were corroborated with a D-(+)-Glucose (Glu) standard curve ($R^2$ = 0.99). The results were expressed as mg Glu $g^{-1}$ extract.

The total protein concentration of the extracts was evaluated using the Bradford method reagents (Sigma-Aldrich, Madrid, Spain) according to the manufacturer's specifications. Briefly, 5 µL of the sample buffer was reacted with 250 µL of the Bradford reagent and incubated for 30 min. Then, the absorbance was measured at 595 nm (BioTeck Synergy HT microplate reader, Winooski, Vermont, USA). The extract solvent was used as blank. BSA (bovine serum albumin) was used as a standard for protein quantification. All the measurements were done in triplicate; the results were expressed as mg BSA $g^{-1}$ extract.

*S.2.6. Chemical characterization by Gas chromatography-mass spectrometry*

Before GC-MS analysis, propolis extracts were derivatized to trimethylsilyl (TMS) ethers to identify the lipids and other volatile compounds. First, 50 µL of a propolis extract (10 mg $mL^{-1}$) was dried under a nitrogen atmosphere. Then, 50 µL of the derivatizing agent (MSTFA) was added, mixed, and heated to 40 °C for 20 min. Finally, 1 µL of the resulting mixture was used for GC–MS analysis (Hrabovski et al., 2012).

The chemical analysis was conducted on a GC-MS (Shimadzu Corporation, Kyoto, Japan) system. The compounds were separated using a Zebron ZB-5Plus GC column (30 m x 250 µm i. D x 0.25 µm, Phenomenex, Torrance, CA, USA), and helium was used as carrier gas at a constant 1 mL min-1 flow rate. A volume of 1.0 µL was injected into the GC with a split mode (split ratio 10). The temperature was programmed to start at 100 °C (1 min) and increase to 250 °C (20 °C $min^{-1}$), where it was held for 2 min. Then, the temperature was increased to 300 (10 °C $min^{-1}$) and held until 25 min. MS parameters were the following: interface temperature, 280 °C; source temperature, 250 °C; mass range, 40–500 m/ z; scan speed, 1428 amu $s^{-1}$. The data analyses were performed using the GCMS solution (ver. 4.45, Shimadzu) software. The compounds were identified using the National Institute of Standards and Technology (NIST) and WILEY libraries with the GC-MS Solution software (Shimadzu, Kyoto, Japan).

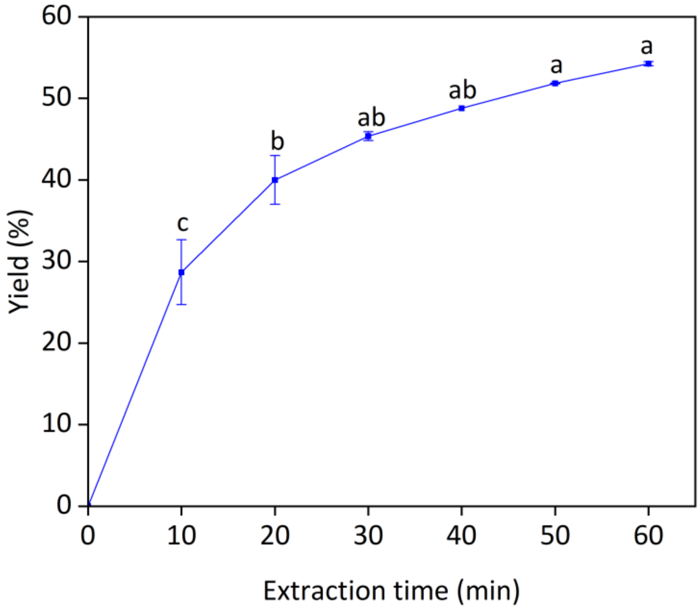


**Figure S1.** SFE kinetic of green propolis at 4 mL $min^{-1}$, 200 bar, 50 ºC, and 20% CPME as cosolvent.

**Table S1.** Experimental design for the full-factorial design (FFD) applied to obtain the optimum process parameters (co-solvent - CPME and temperature) of the SFE of bioactive molecules from green propolis as first step of biorefinery process. Extraction pressure 20 MPa and flow 4mL min$^{-1}$ constants in all experiments.

| **CPME (%)** | **Temp. (°C)** | **Yield (%)** | **TPC (mg GAE g$^{-1}$)** | **ABTS (µmol TE g$^{-1}$)** | **DPPH (µmol TE g$^{-1}$)** | **LOX (mg QE g$^{-1}$)** |
|---|---|---|---|---|---|---|
| 10 | 40 | 34 ± 3 | 118 ± 42 | 1488 ± 76 | 579 ± 24 | 28 ± 4 |
| 10 | 50 | 30 ± 2 | 111 ± 7 | 1397 ± 69 | 584 ± 39 | 22 ± 12 |
| 10 | 60 | 30 ± 3 | 104 ± 2 | 1346 ± 35 | 599 ± 27 | 31 ± 6 |
| 20 | 40 | 36 ± 2 | 129 ± 5 | 1622 ± 94 | 597 ± 80 | 33 ± 3 |
| 20 | 50 | 43.1 ± 0.5 | 114 ± 9 | 2149 ± 158 | 620 ± 46 | 24 ± 7 |
| 20 | 60 | 51 ± 5 | 132.4 ± 0.7 | 2344 ± 188 | 639 ± 17 | 27 ± 6 |
| 30 | 40 | 48 ± 7 | 154 ± 9 | 2425 ± 239 | 644 ± 9 | 26 ± 5 |
| 30 | 50 | 42 ± 2 | 130 ± 8 | 1434 ± 57 | 618 ± 31 | 21 ± 6 |
| 30 | 60 | 59.6 ± 0.1 | 199 ± 26 | 2703 ± 143 | 637.8 ± 0.4 | 14 ± 1 |
| **SOX-Hex** | | 18.9 ± 0.7 | 110 ± 8 | 1408 ± 102 | 663 ± 3 | 14 ± 5 |

Experimental data are expressed as the mean ± SD (n = 2

 https://doi.org/10.3390/molecules30010189

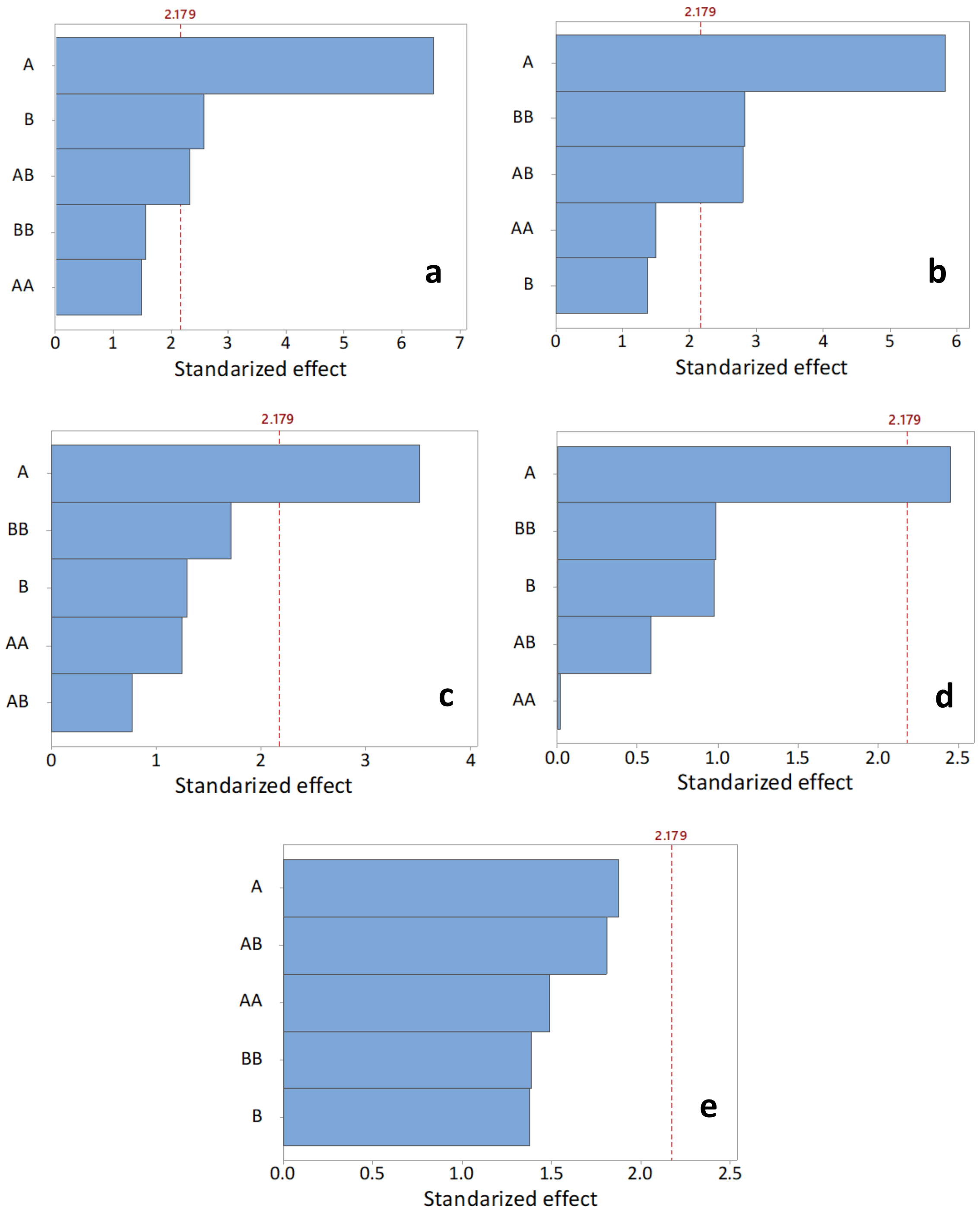


**Figure S2.** Pareto chart of standardized effects (**a**) yield, (**b**) TPC, (**c**) ABTS, (**d**) DPPH, (**e**) LOX. Factors (A) co-solvent, (B) temperature. $\alpha = 0.05$.

**Table S2.** ANOVA of the fitted Full Factorial model for SFE yield (%) from green propolis.

| Source | Sum of Squares | df | Mean square | F-value | p-value |
|---|---|---|---|---|---|
| A: Co-solvent (%) | 1056.75 | 1 | 1056.75 | 42.99 | 0.000* |
| B: Temperature (°C) | 162.14 | 1 | 162.14 | 6.6 | 0.025* |
| $A^2$ | 113.78 | 1 | 56.89 | 2.31 | 0.141 |
| $B^2$ | 54 | 1 | 54 | 2.2 | 0.164 |
| AB | 132.44 | 1 | 132.44 | 5.39 | 0.039* |
| Lack of fit | 190.94 | 3 | 63.65 | 5.51 | 0.02* |
| Pure Error | 104.04 | 9 | 11.56 | | |
| Total Sum of Squares | 1760.09 | 17 | | | |

Significant effects at p = 0.05 are marked with an asterisk(*).

**R-squared =** 83.24%

**R-squared adjusted ($R^2$) =** 76.26%

**Standard Error of the Regression =** 4.95798

**Equation of the fitted model for Yield (%):**

Yield (%) = 127.1 + 0.37 * A - 4.31 * B - 0.04 * $A^2$ + 0.04 * $B^2$ + 0.04 * A * B

**Table S3.** ANOVA of the fitted Full Factorial model for TPC (mg GAE $g^{-1}$) from green propolis.

| Source | Sum of Squares | df | Mean square | F-value | p-value |
|---|---|---|---|---|---|
| A: Co-solvent (%) | 7344.8 | 1 | 7344.8 | 34 | 0.000* |
| B: Temperature (°C) | 408.6 | 1 | 408.6 | 1.89 | 0.194 |
| $A^2$ | 489.4 | 1 | 489.4 | 2.27 | 0.158 |
| $B^2$ | 1729.2 | 1 | 1729.2 | 8 | 0.015* |
| AB | 1697.1 | 1 | 1697.1 | 7.86 | 0.016* |
| Lack of fit | 1545.3 | 3 | 515.1 | 4.43 | 0.036* |
| Pure Error | 1047.2 | 9 | 1047.2 | 116.4 | |
| Total Sum of Squares | 14261.5 | 17 | | | |

Significant effects at p = 0.05 are marked with an asterisk(*).

**R-squared** = 81.82%

**R-squared adjusted ($R^2$)** = 74.25%

**Standard Error of the Regression** = 14.6985

**Equation of the fitted model for TPC (mg GAE $g^{-1}$):**

TPC (mg GAE $g^{-1}$) = 742 - 9.23 * A - 23.12 * B + 0.11 *$A^2$ + 0.21 * $B^2$ + 0.15 * A * B

**Table S4.** ANOVA of the fitted Full Factorial model for ABTS (μMol TE $g^{-1}$) from green propolis.

| Source | Sum of Squares | df | Mean square | F-value | p-value |
|---|---|---|---|---|---|
| A: Co-solvent (%) | 1811715 | 1 | 1811715 | 12.38 | 0.004* |
| B: Temperature (°C) | 246132 | 1 | 246132 | 1.68 | 0.219 |
| $A^2$ | 230023 | 1 | 230023 | 1.57 | 0.234 |
| $B^2$ | 431351 | 1 | 431351 | 2.95 | 0.112 |
| AB | 88024 | 1 | 88024 | 0.6 | 0.453 |
| Lack of fit | 1594683 | 3 | 531561 | 29.54 | 0.000* |
| Pure Error | 161940 | 9 | 17993 | | |
| Total Sum of Squares | 4563868 | 17 | | | |

Significant effects at p = 0.05 are marked with an asterisk(*).

**R-squared =** 61.51%
**R-squared adjusted ($R^2$) =** 45.47%
**Standard Error of the Regression =** 382.603

**Equation of the fitted model for TPC (μMol TE $g^{-1}$):**
ABTS (μMol TE $g^{-1}$) $= 8626 + 82 * A - 335 * B - 2.40\ A^2 + 3.28\ B^2 + 1.05 * A * B$

**Table S5.** ANOVA of the fitted Full Factorial model for DPPH (μMol TE $g^{-1}$) from green propolis.

| Source | Sum of Squares | df | Mean square | F-value | p-value |
|---|---|---|---|---|---|
| A: Co-solvent (%) | 6411.3 | 1 | 6411.27 | 6 | 0.031* |
| B: Temperature (°C) | 1022.1 | 1 | 1022.13 | 0.96 | 0.347 |
| $A^2$ | 0.3 | 1 | 0.34 | 0 | 0.986 |
| $B^2$ | 1048.7 | 1 | 1048.75 | 0.98 | 0.341 |
| AB | 362.7 | 1 | 362.68 | 0.34 | 0.571 |
| Lack of fit | 1096.3 | 3 | 365.43 | 0.28 | 0.838 |
| Pure Error | 11722.3 | 9 | 1302.48 | | |
| Total Sum of Squares | 21663.8 | 17 | | | |

Significant effects at p = 0.05 are marked with an asterisk(*).

**R-squared =** 40.83%
**R-squared adjusted ($R^2$) =** 16.17%
**Standard Error of the Regression =** 32.68

**Equation of the fitted model for TPC (μMol TE $g^{-1}$):**
DPPH (μMol TE $g^{-1}$) $= 844 + 5.80 * A – 13.90 * B – 0.003\ A^2 + 0.16\ B^2 – 0.07 * A * B$

**Table S6.** ANOVA of the fitted Full Factorial model for LOX (mg QE $g^{-1}$) from green propolis. 68

| Source | Sum of Squares | df | Mean square | F-value | p-value |
|---|---|---|---|---|---|
| A: Co-solvent (%) | 121.38 | 1 | 121.38 | 3.54 | 0.084 |
| B: Temperature (°C) | 65.46 | 1 | 65.46 | 1.91 | 0.192 |
| $A^2$ | 76.88 | 1 | 76.88 | 2.24 | 0.16 |
| $B^2$ | 66.51 | 1 | 66.51 | 1.94 | 0.189 |
| AB | 112.73 | 1 | 112.73 | 3.29 | 0.095 |
| Lack of fit | 67.79 | 3 | 22.6 | 0.59 | 0.636 |
| Pure Error | 343.82 | 9 | 38.2 | | |
| Total Sum of Squares | 854.58 | 17 | | | |

Significant effects at p = 0.05 are marked with an asterisk(*).

**R-squared =** 51.83%
**R-squared adjusted ($R^2$) =** 31.77%
**Standard Error of the Regression =** 5.86

**Equation of the fitted model for TPC (μMol TE $g^{-1}$):**
LOX (mg QE $g^{-1}$) = $90.1 + 3.31 * A – 3.56 * B – 0.04 A^2 + 0.04 B^2 – 0.04 * A * B$

Table S7. Tentatively identified compounds by GC-MS/MS analysis from SFE extracts of green propolis extracts. 79

| Peak | Ret. time (min) | Family | Tentative identification | Formula | Match Factor (%) | Monoisotopic mass | Main Fragments (m/z) |
|---|---|---|---|---|---|---|---|
| 1 | 5.694 | Phenolic | Benzenepropanoic acid | C12H18O2Si | 94 | 222 | 75, 104, 207, 222 |
| 2 | 5.849 | Terpenoid | Trans-Caryophyllene | C15H24 | 95 | 204 | 41, 69, 93, 133 |
| 3 | 5.976 | Terpenoid | Trans-Caryophyllene isomer | C15H24 | 90 | 204 | 41, 69, 93, 133 |
| 4 | 6.339 | Terpenoid | Phenol, 2,6-di-tert-butylphenol | C15H24O | 84 | 220 | 205, 57 |
| 5 | 6.453 | Terpenoid | α-Copaene | C15H24 | 78 | 204 | 105, 119, 161, 41 |
| 6 | 6.618 | Terpenoid | Nerolidol isomer | C15H26O | 98 | 222 | 69, 41, 93 |
| 7 | 6.857 | Terpenoid | Spathulenol | C15H24O | 89 | 220 | 43, 93, 91, 119 |
| 8 | 6.912 | Terpenoid | Caryophyllene oxide | C15H24O | 89 | 220 | 43, 55, 79, 93, 121 |
| 9 | 7.314 | Terpenoid | Trans, trans-Farnesol | C18H34OSi | 86 | 294 | 41, 69, 73, 93, 107 |
| 10 | 7.474 | Terpenoid | Pseduosarsasapogenin-5,20-dien | C28H44O3 | 75 | 428 | 41, 55, 73, 107 |
| 11 | 7.65 | Terpenoid | Trans, trans-Farnesol isomer | C18H34OSi | 81 | 294 | 41, 69, 93 |
| 12 | 7.772 | Phenolic | Hydrocinnamic acid | C15H26O3Si2 | 85 | 310 | 45, 73, 163, 179, 205 |
| 13 | 8.733 | Phenolic | Cinnamic acid | C15H24O3Si2 | 93 | 308 | 73, 219, 249, 293, 308 |
| 14 | 9.076 | Terpenoid | Pentitol | C17H42D2O4Si | 78 | 424 | 73, 105, 147 |
| 15 | 9.255 | Fatty acid | Hexadecanoic acid (palmitic acid) | C19H40O2Si | 92 | 328 | 43, 73, 117, 132, 145 |
| 16 | 9.924 | Phenolic | 2-Hydroxyoctanoic acid | C11H24O3Si | 71 | 232 | 55, 73, 97, 129 |
| 17 | 10.027 | Phenolic | Phosphonic acid | C12H23O3PSi2 | 68 | 302 | 45, 73, 287, 302 |
| 18 | 10.626 | Fatty acid | Octadecanoic acid (stearic acid) | C21H44O2Si | 92 | 356 | 43, 73, 117, 145 |
| 19 | 11.13 | Fatty acid | 9,12-Octadecadienoic acid (linoleic acid) | C21H40O2Si | 76 | 352 | 55, 75, 95, 121 |

| | | | | | | | |
|---|---|---|---|---|---|---|---|
| **20** | 11.518 | Fatty acid | α-linolenic acid | C21H40O2Si | 76 | 352 | 55, 75, 95, 121 |
| **21** | 11.633 | Fatty acid | α-linolenic acid isomer | C21H38O2Si | 77 | 350 | 41, 73, 95, 121 |
| **22** | 11.838 | Fatty acid | α-linolenic acid isomer 2 | C21H38O2Si | 77 | 350 | 41, 73, 95, 121 |
| **23** | 11.929 | Fatty acid | 9,12-Octadecadienoic acid isomer 2 | C21H38O2Si | 77 | 350 | 41, 73, 95, 121 |
| **24** | 12.06 | Fatty acid | 11-Eicosenoic acid (omega 9) | C21H40O2Si | 75 | 352 | 55, 73, 95, 117 |
| **25** | 12.239 | Fatty acid | Pentanoic acid | C8H16O3Si | 78 | 188 | 43, 73, 101, 129 |
| **26** | 12.661 | Terpenoid | Farnesol | C18H34OSi | 76 | 294 | 41, 69, 73, 109 |
| **27** | 13.087 | Fatty acid | Pentanoic acid (valeric acid) isomer | C8H16O3Si | 73 | 188 | 41, 43, 73, 129 |
| **28** | 13.521 | Fatty acid | Hexadecanoic acid (monopalmitin) | C25H54O4Si2 | 91 | 474 | 43, 57, 73, 103, 129 |
| **29** | 13.777 | Terpenoid | Myrtenol | C10H18O | 91 | 154 | 121, 107, 79, 77, 58 |
| **30** | 14.153 | Fatty acid | 5-Eicosene | C20H40 | 82 | 280 | 55, 69, 83, 97 |
| **31** | 14.596 | Terpenoid | Tetrahydro linalool | C10H22O | 69 | 158 | 43, 59, 73 |
| **32** | 14.743 | Phenolic | 1,3-Benzenedicarboxylic acid | C24H38O4 | 79 | 390 | 57, 70, 112, 167 |
| **33** | 16.975 | Fatty acid | Tetradecanedioic acid | C26H54O4Si | 75 | 486 | 75, 115, 129, 429 |
| **34** | 18.097 | Phenolic | Trans-Aconitic acid | C24H48O6Si3 | 63 | 516 | 73, 115, 327, 459 |
| **35** | 19.094 | Fatty acid | Tetracosanoic acid (lignoceric acid) | C27H56O2Si | 86 | 440 | 43, 73, 117, 132 |
| **36** | 19.389 | Fatty acid | 1,19-Eicosadiene | C20H38 | 89 | 278 | 55, 69, 82, 96 |
| **37** | 21.069 | Sterol | Ergost-25-ene-3,5,6,12-tetrol | C28H48O4 | 76 | 448 | 55, 69, 81, 95, 97 |
| **38** | 21.46 | Fatty acid | Cis-5,8,11-Eicosatrienoic acid | C23H42O2Si | 75 | 378 | 55, 73, 95, 97, 109 |
| **39** | 22.047 | Fatty acid | Decanoic acid | C13H27FO2Si | 76 | 262 | 55, 73, 117, 145 |
| **40** | 23.891 | Terpenoid | Globulol | C15H26O | 84 | 222 | 43, 69, 93, 109 122 |

81
